# Coagulation derangement and risk factors for valve thrombosis following transcatheter aortic valve implantation

**DOI:** 10.1136/openhrt-2020-001496

**Published:** 2021-06-14

**Authors:** Tiffany Patterson, Harriet Hurrell, Jack Lee, Giulia Esposito, Utkarsh Dutta, Julia Grapsa, Nicholas Aroney, Fiyyaz Ahmed-Jushuf, Christopher Allen, Ronak Rajani, Rebecca Preston, Christopher Young, Gianluca Lucchese, Kiran Parmar, Beverley Hunt, Bernard D Prendergast, Simon R Redwood

**Affiliations:** 1 Cardiovascular Division, School of Life Science and Medicine, King's College London, London, UK; 2 Division of Biomedical Engineering and Imaging Sciences, King's College London, London, UK; 3 Cardiovascular Department, Guy's and St Thomas' NHS Foundation Trust, London, UK; 4 Department of Radiology and Cardiac CT, Guy's and St Thomas' NHS Foundation Trust, London, UK; 5 Department of Haematology and Thrombosis, Guy's and St Thomas' NHS Foundation Trust, London, UK

**Keywords:** aortic valve stenosis, heart valve diseases, transcatheter aortic valve replacement

## Abstract

**Aims:**

Durability of transcatheter aortic valve implantation (TAVI) is key to its expansion. We sought to identify incidence of valve thrombosis and predictors of valve thrombosis in our single centre with associated coagulation testing pre-TAVI and post-TAVI.

**Methods and results:**

This single-centre observational study comprised patients undergoing transfemoral TAVI discussed in the Heart Team meeting. Patients were followed up with echocardiography at 120 days to identify incidence of elevated transvalvular gradient and multivariable analysis was performed to identify factors associated with an increased odds of developing valve thrombosis. In addition, 11 patients underwent baseline, day 1 and day 120 post-TAVI coagulation testing. Between August 2017 and August 2019, 437 consecutive patients underwent transfemoral TAVI. Of these patients, 207/437 (47.4%) had 3-month follow-up echo data available and were analysed. Of these patients, 26/207 (12.6%) had elevated transvalvular gradients. These patients tended to be younger (80±14 vs 83±6 years; p=0.047) with a lower ejection fraction (49±13 vs 54%±11%; p=0.021), with a greater proportion of the population experiencing atrial fibrillation (14/21, 54% vs 68/181, 38%; p=0.067). Following multivariable analysis, there remained a trend towards higher eccentricity index associated with elevated gradients. Baseline (pre-TAVI) elevation of thrombin antithrombin levels (56±63; reference range 1.0–4.1 ng/L) and PF 1+2 (791±632; reference range 69–229 ng/mL) normalised at 120 days post-TAVI

**Conclusion:**

This study demonstrated that in the cohort of patients undergoing transfemoral TAVI in our centre: younger age, poor ejection fraction, atrial fibrillation and increased baseline eccentricity of the aortic valve annulus were present to a greater extent in patients exhibiting elevated transvalvular gradients at 3-month follow-up. Further work is required to delineate the extent of coagulation derangement and confirm predictors of thrombosis.

**Key questions**

**What is already known about this subject?**

- Baseline coagulation derangement has not been well characterised in the aortic stenotic population and little is known regarding the real-world incidence of transcatheter aortic valve implantation (TAVI) leaflet thrombosis.

**What does this study add?**

- This study demonstrated that in the cohort of patients undergoing transfemoral TAVI in our centre: younger age, poor ejection fraction, atrial fibrillation and increased baseline eccentricity of the aortic valve annulus were present to a greater extent in patients exhibiting elevated transvalvular gradients.

**How might this impact on clinical practice?**

- Understanding predictors of valve thrombosis will enable us to tailor anticoagulation therapy appropriately post-TAVI.

## Introduction

Transcatheter aortic valve implantation (TAVI) has become an important alternative to surgical aortic valve replacement (sAVR). TAVI demand is expected to increase exponentially with an ageing population. Early clinical outcomes are at least equivalent, if not superior to sAVR, regardless of risk score.^1^ Durability is key if TAVI is to expand into the younger population. There has been recent concern as to the detection of TAVI leaflet thrombosis in up to 15% of patients at follow-up. TAVI thrombosis has been reported to lead to early valve failure presenting as elevated transvalvular gradients and dyspnoea, thromboembolism and stroke. Low cardiac output states can reduce transvalvular blood flow thus promoting stasis. The impact of low flow is well demonstrated in mitral prostheses, which carry a threefold increased risk of thrombosis in comparison with aortic prostheses.^2^ Artificial valve leaflets, local tissue injury during the procedure and persistence of the native calcified valve leaflets increase tissue factor release thereby inducing local activation of the extrinsic coagulation pathway. This is in contrast to the normal endothelium, which produce an antithrombotic milieu. Chronic kidney disease, anaemia and obesity, which occur more commonly in the high-risk TAVI population are also associated with prothrombotic states.^3^ However, baseline coagulation derangement has not been well characterised in the aortic stenotic population and little is known regarding the real-world incidence of TAVI thrombosis. Furthermore, routine anticoagulation post-TAVI has been associated with harm. It is, therefore, essential to determine risk factors and contributors to valve thrombosis. In this observational study, consecutive patients undergoing transfemoral TAVI were examined in our single centre over a 2-year period, with assessment of baseline characteristics and follow-up echocardiography to identify the incidence of elevated transvalvular gradient at 3 months compared with baseline postprocedure with a view to determining predictors of valve thrombosis.^4^ In addition, we performed coagulation tests periprocedurally and at 3 months in a subset of patients to establish baseline and postprocedural coagulation derangement in this cohort.

## Methods

### Study design and population

This prospective observational study was conducted at a single centre: St Thomas Hospital, London, UK. This study included all consecutive patients that underwent successful transfemoral TAVI procedure between August 2017 and August 2019. Prior to TAVI implantation, all patients were discussed in a dedicated Heart Team Meeting.

To identify baseline coagulation derangement in the aortic stenotic population and the duration of coagulation derangement post TAVI, a random sample of 11 patients awaiting TAVI was examined. Blood test time points were as follows: on admission prior to TAVI implantation, day 1 post-TAVI and at 3 months post-TAVI. The coagulation testing comprised haemoglobin level (Hb), mean corpuscular volume, white cell count (WCC), platelet count (PLT), thrombin antithrombin (TAT), D-dimer, lactate dehydrogenase (LDH), fibrinogen, prothrombin factors 1+2 (PF1 +2), international normalised ratio, activated prothrombin time. TAT and PF1 +2 were selected as they represent thrombin generation. D-dimer is an indicator of thrombosis and endogenous fibrinolytic activity. LDH is a marker of haemolysis.

In order to identify the incidence of elevated transvalvular gradients, follow-up echocardiography was performed 3 months postprocedure and compared with the immediate postprocedure echocardiogram. Patients were deemed to have leaflet thrombosis based on both clinical assessment and fulfilment of the following criteria: elevated mean bioprosthetic valve gradient of greater than 10 mm Hg at 3 months compared with the immediate post-TAVI echocardiogram (structural valve deterioration defined according to ESC guidance) with accompanied restricted leaflet motion on echocardiogram at 3 months follow-up.^4^ For the purposes of analysis at 3 months, patients were classified according to the presence or absence of elevated transvalvular gradients compared with the immediate post-TAVI echocardiogram. Baseline characteristics collected included cardiovascular risk factors, presence of antiplatelet and/or anticoagulation therapy, New York Heart Association (NYHA) Class, ejection fraction and pre-TAVI CT parameters including annulus area and eccentricity index (defined as the major annulus diameter/minor annulus diameter as a percentage).

### Statistical analysis

In reference to baseline characteristics and potential risk factors associated with elevated transvalvular gradients, categorical data were presented as counts and percentages, and comparison between groups (presence or absence of elevated transvalvular gradients) performed using χ^2^ test or Fisher’s exact test; continuous data of normal distribution were presented as mean±SD and comparison between groups performed using independent t-test. Statistical comparison of serial coagulation testing (pre-TAVI, post-TAVI day 1 and day 120) was performed using one-way analysis of variance; thereafter, analysis of specific sample pairs (paired differences) was performed compared using paired t-test.

Univariate analysis was used to identify potential predictors of elevated transvalvular gradient. Variables associated with a p<0.05 were then included in the multivariate analysis. The variance inflation factor was used to determine colinearity using standardised cut-offs. To determine clinical predictors of valve thrombosis, the covariates entered into the equation were: age, presence of atrial fibrillation, ejection fraction, postprocedure echo gradient and the eccentricity index. Adjusted ORs (OR) with 95% CIs are reported. All p values were two sided with a significance threshold p<0.05. Statistical analysis was performed using SPSS V.24.0 (IBM).

## Results

Between August 2017 and August 2019, 437 consecutive patients underwent transfemoral TAVI. Of these patients, 207/437 (47.4%) had 3 months follow-up echo data available and were analysed. Of these patients, 26/207 (12.6%) had elevated transvalvular gradients. Baseline characteristics including demographics, cardiovascular risk factors, NYHA class at presentation and ejection fraction are provided in table 1. Patients with an elevated transvalvular gradient at follow-up (mean gradient >10 mmHg compared with immediately post-TAVI) tended to be younger (80±14 vs 83±6 years; p=0.047) with a lower ejection fraction (49±13 vs 54%±11%; p=0.021), with a greater proportion of the population experiencing atrial fibrillation (14/21, 54% vs 68/181, 38%; p=0.067) when compared with those without elevated gradients. Pre-TAVI CT parameters and immediate post-TAVI echo parameters are shown in table 2. Patients with an elevated transvalvular gradient at follow-up demonstrated higher eccentricity of the annulus (21±4 vs 18%±6%; p=0.019) and lower echo-derived bioprosthetic aortic valve gradients (9.4±4.5 vs 12.5±6; p=0.003) and echo-derived peak bioprosthetic aortic valve velocity (1.9±0.5 vs 2.3±0.5 m/s; p<0.001). There were no other observed between the two groups.

**Table 1:** Baseline characteristics

Variable	Elevated gradient n=26	No gradient change n=181	P value	Total
Age	80±14	83±6	0.047*	83±8
Height (cm)	165±10	166±10	0.835	166±10
Weight (kg)	76±18	75±16	0.942	75±16
Sex (M)	14 (54)	94 (52)	0.855	108 (51)
Hypertension	21 (81)	132 (73)	0.494	156 (74)
Hyperlipidaemia	17 (65)	93 (51)	0.209	112 (53)
Diabetes	21 (81)	44 (24)	0.559	50 (24)
Smoker	10 (39)	70 (39)	0.755	82 (39)
Coronary disease	15 (58)	81 (45)	0.301	99 (47)
Previous MI	5 (19)	33 (18)	0.985	41 (19)
Previous PCI	4 (15)	36 (20)	0.576	40 (19)
Previous CABG	5 (19)	26 (14)	0.556	31 (15)
Anticoagulation	11 (42)	69 (38)	0.698	80 (38)
Antiplatelets	13 (50)	86 (48)	0.832	103 (49)
Atrial fibrillation	14 (54)	68 (38)	0.067	82 (39)
PPM preprocedure	1 (4)	22 (12)	0.197	24 (11)
PVD	2 (8)	9 (5)	0.594	11 (5)
TIA/Stroke	4 (16)	28 (16)	0.964	33 (16)
Renal disease eGFR <30	10 (39)	47 (26)	0.188	58 (28)
Chronic lung disease	4 (15)	51 (28)	0.163	57 (27)
NYHA Class				
I	2 (8)	13 (7)	0.782	16 (8)
II	4 (15)	43 (24)		48 (23)
III	13 (50)	78 (43)		93 (44)
IV	2 (8)	18 (10)		20 (10)
Ejection fraction	49±13	54±11	0.021*	54±11

This table describes the presence of baseline characteristic per group: elevated gradient at follow-up versus no elevated gradient and a p value for comparison of the groups using independent t-test (for continuous) or χ2 tests (for categorical variables). Baseline characteristic of the entire cohort are also summarised in the ‘Total’ column. Data are presented as n (%) or mean±SD.

*P<0.05 (statistical significance)

CABG, coronary artery bypass graft surgery; eGFR, estimatedglomerular filtration rate; M, male; MI, myocardial infarction; NYHA, New York Heart Association classification of breathlessness; PCI, percutaneous coronary intervention; PPM, permanent pacemaker; PVD, peripheral vascular disease; TIA, transient ischaemic attack.

**Table 2:** Imaging characteristics

Variable	Elevated gradient n=26	No gradient change n=181	P value	Total
MG echo day 1 post-TAVI, mm Hg	9.4±4.5	12.5±6	0.003*	12.2±6.1
Velocity echo day 1 post-TAVI m/s	1.9±0.5	2.3±0.5	<0.001*	2.3±0.5
CT-derived annulus area (pre-TAVI) mm^2^	449±77	454±80	0.748	452±79
CT-derived annulus perimeter (pre-TAVI) mm	77±7	77±8	0.716	77±8
Major annulus diameter mm	27±2	27±3	0.731	27±3
Eccentricity index %	21±4	18±6	0.019*	19±6
Sinotubular junction (major)	29.0±3.6	29.6±4.0	0.640	29.5±3.9
Sinus of Valsalva (major)	32.4±3.2	33.0±4	0.449	32.9±3.9

This table describes and compares preprocedural CT parameters and also the initial immediate postprocedural echo on day 1 post-TAVI between groups: elevated gradient at follow-up versus no elevated gradient and a p value for comparison of the groups using independent t-test (for continuous variables). The entire cohort are also summarised in the ‘Total’ column. CT-derived annulus area and perimeter refer to the aortic valve annulus.

*P<0.05 (statistical significance)

MG, mean gradient; TAVI, transcatheter aortic valve implantation.

Significant variables were used to perform multivariable logistic regression analysis. The outcomes are reported in table 3. Multivariable analysis did not demonstrate an association between ejection fraction, mean gradient, peak velocity, atrial fibrillation and elevated transvalvular gradients. However, there remained a trend towards older age being negatively associated with elevated transvalvular gradients (OR 0.925, 95% CI 0.836 to 1.024; p=0.132). There was also a trend toward higher eccentricity index being associated with elevated transvalvular gradients (OR 1.107, 95% CI 0.983 to 1.247; p=0.095).

**Table 3:** Results of the multivariate analysis

Variable	OR	95% CI	P value
Age years	0.925	0.836 to 1.024	0.132
Atrial fibrillation	1.028	0.283 to 3.739	0.967
Ejection fraction %	0.972	0.921 to 1.027	0.312
Post-TAVI* mean gradient, mm Hg	0.960	0.719 to 1.280	0.779
Post-TAVI* peak velocity m/s	0.288	0.016 to 5.056	0.394
Eccentricity Index	1.107	0.983 to 1.247	0.095

This table reports the odds of developing elevated transvalvular gradient per risk factor. Data are reported as ORs and 95% CIs.

*Performed at day 1 or day 2 immediately post-TAVI and prior to discharge.

TAVI, transcatheter aortic valve implantation.

**Figure 1:**
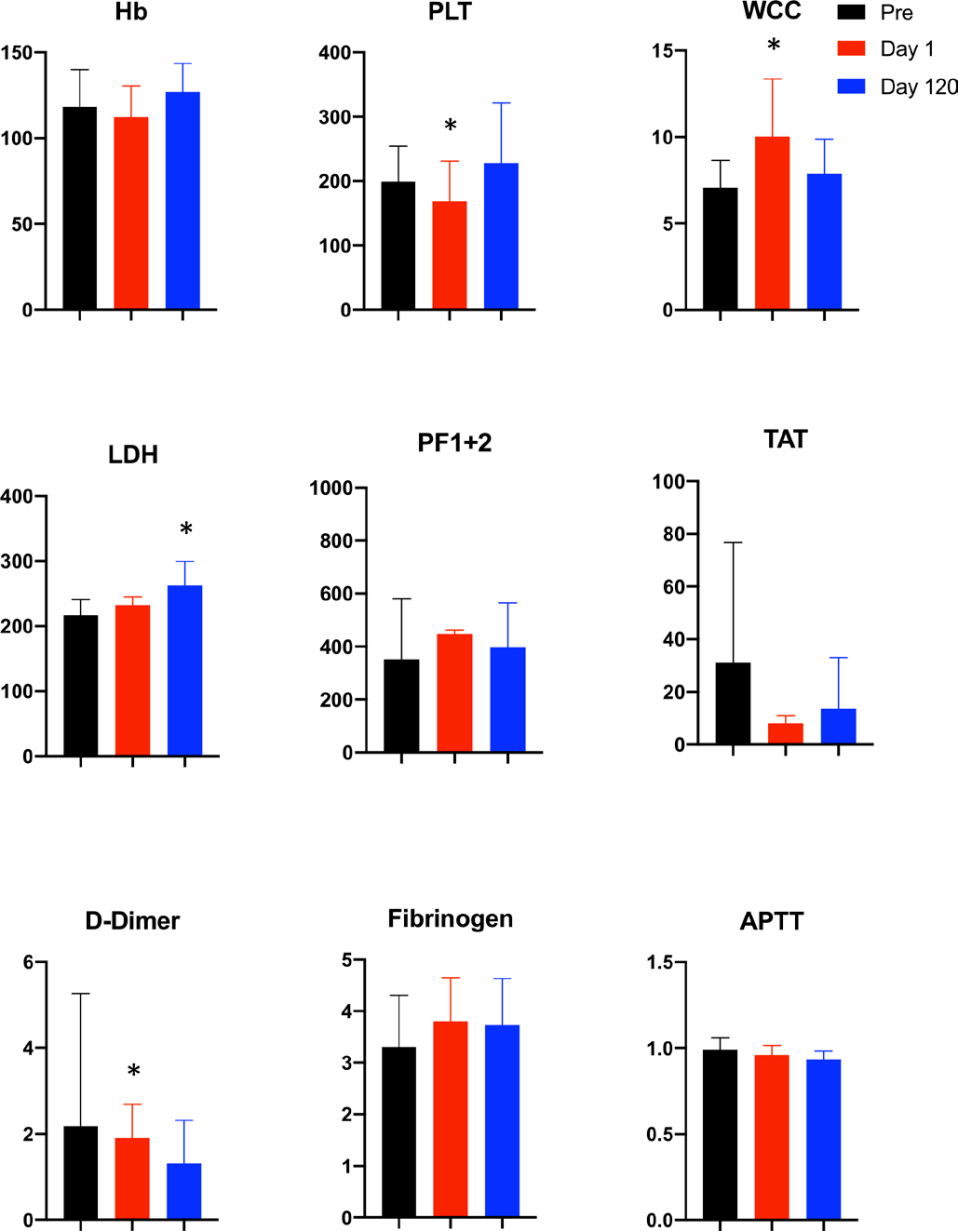
Coagulation testing pre-TAVI and at day 1 and 120 post-TAVI. From left to right: haemoglobin (Hb), platelet (PLT), white cell count (WCC), lactate dehydrogenase (LDH), prothrombin factors 1+2 (PF 1+2), thrombin anti-thrombin (TAT), d-dimer, fibrinogen, activated partial thromboplastin clotting time. Blood tests were performed in 11 patients pre-TAVI (black column) and day1 (red column) and day 120 (blue column) post-TAVI. *Indicates significant change compared with pre-TAVI. Reference ranges: Hb 120–150, pLT 150–400, WCC 4.0–10.0, LDH 135–214, PF 1+2 69.0–229.0, TAT 1.0–4.10 D-dimer 0.00–0.55, fibrinogen 1.7–3.9, APTT 0.8–1.2. APTT, activated prothrombin time; TAVI, transcatheter aortic valve implantation.

The results of serial coagulation testing are presented in table 4 and depicted graphically in figure 1. Of the 11 patients, serial blood testing was performed in nine patients (two patients could not perform follow-up tests due to limited mobility). Coagulation testing demonstrated baseline (pre-TAVI) elevation of TAT levels (56±63; reference range 1.0–4.1 ng/L), and PF 1+2 (791±632; reference range 69–229 ng/mL) which normalised by 3 months, however, this did not reach significance. As expected, there was a postprocedure decrease in Hb (112±18.2 vs 118±21.5; p=0.082) and platelets (169±52.2 vs 199±54.9; p=0.009) with an increase in WCC (10.0±3.3 vs 7.1±1.6, p=0.003) and D-dimer (1.86±0.79 vs 0.49±0.27, p=0.046) levels. LDH remained slightly elevated 3 months post-TAVI.

**Table 4:** Coagulation testing in 11 patients at baseline, day 1 and day 120 post-TAVI

Coagulation markers	A. Pre-TAVI	B. day 1 post-TAVI	C. day 120 post-TAVI	P value A vs B	P value A vs C
Haemoglobin (120–150 g/L)	118±21.5	112±18.2	127±16.4	0.082	0.330
Platelets (150–400×10^9^/L)	199±54.9	169±52.2	227±93.6	0.009*	0.406
White cell count (4–10×10^9^/L)	7.1±1.6	10.0±3.3	7.9±2.0	0.003*	0.315
LDH (135–214 U/L)	224±18	232±13	243±31	0.232	0.015*
PF 1+2 (69–229 ng/mL)	791±632	451±11.1	401±11.1	0.453	0.352
TAT (1.0–4.1 ng/mL)	56±63	8.1±2.9	3.9±0.3	0.332	0.124
Fibrinogen (1.7–3.9 s)	3.6±0.8	3.8±0.8	4.3±0.9	–	0.110
D-Dimer (0–0.55 ng/mL)	0.49±0.27	1.86±0.79	0.87±0.47	0.046*	0.465
APTT (0.8–1.2 ratio)	0.96±0.05	0.96±0.05	0.90±0.00	–	0.465

This table demonstrates a random sample of 11 patients who attended for TAVI. This demonstrates that a baseline derangement in clotting exists in all patients with aortic stenosis, this is further exacerbated by the procedure, and tends to settle at day 120 following valve endothelialisation. Reference ranges are provided in brackets. Data are presented as mean±SD.

*P<0.05 (statistical significance)

LDH, lactate dehydrogenase; PF 1+2, prothrombin factors 1+2; TAT, thrombin antithrombin; TAVI, transcatheter aortic valve implantation.

## Discussion

This study demonstrated that in the cohort of patients undergoing transfemoral TAVI in our centre: younger age, poor ejection fraction, atrial fibrillation and increased baseline eccentricity of the aortic valve annulus were present to a greater extent in patients exhibiting elevated transvalvular gradients at 3 months follow-up. Multivariable analysis also demonstrated a trend towards an increased probability of elevated transvalvular gradients in younger patients with a higher eccentricity index. This study also demonstrated a baseline derangement coagulation tests in patients with aortic stenosis undergoing TAVI with near normalisation of these coagulation factors at 3 months following the TAVI procedure.

Previous work has demonstrated a potential association between smoking, diabetes and valve thrombosis.^5^ These factors have been previously shown to be associated with a prothrombotic state. We were unable to demonstrate a similar association, but this may have been due to smaller numbers in our study cohort. Prior work has also demonstrated post-TAVI eccentricity to be associated with valve thrombosis previously.^5^ This is similar to our findings although we measured eccentricity preprocedure but it would be reasonable to conclude that the two are related with preprocedure eccentricity likely to influence TAVI valve expansion in the presence of calcification. We are, however, limited by the absence of post-TAVI CT assessment to confirm this association. Patients with a lower ejection fraction are likely to exhibit slower flow across the TAVI valve, which is a known factor in the triad of thrombosis. Interestingly, lower postprocedural mean gradient was also associated with thrombosis, however, this is more likely to be a reflection of reduced left ventricular function.

Baseline coagulation derangement was identified in patients pre-TAVI. This is likely secondary to tissue factor release from the native valve leaflets which can persist post-TAVI. PF 1+2 and TAT levels are both markers of thrombin generation, both of which were elevated preprocedure and these tended to normalise by 3 months, which could reflect valve endothelialisation. The increase in LDH post-TAVI follows a periprocedural drop in Hb and the raised D-dimer levels is due to the procedural insult and activation of the clotting cascade. Postmortem studies have suggested endothelialisation of the TAVI leaflets at 120 days, however, our results would suggest that valve endothelialisation is multifactorial and varies between individuals.

The recently published trial of rivaroxaban following TAVI demonstrated that patients without an established indication for oral anticoagulation experienced a higher risk of death or thromboembolic complications and bleeding than an antiplatelet-based strategy.^6^ This highlights that routine use of anticoagulation may cause harm but supports the need to identify predictors of valve thrombosis.

### Limitations

This was a single-centre study. The biomarker screening arm was limited by its small size. Post-TAVI CT scanning was not performed and would have added further useful information.

## Conclusion

This study demonstrated that in the cohort of patients undergoing transfemoral TAVI in our centre: younger age, poor ejection fraction, atrial fibrillation and increased baseline eccentricity of the aortic valve annulus were present to a greater extent in patients exhibiting elevated transvalvular gradients at 3-month follow-up. Further work is essential to examine the extent of coagulation derangement and confirm predictors of thrombosis.

## Data Availability

All data relevant to the study are included in the article or uploaded as online supplemental information.

## References

[1] Mack MJ, Leon MB, Thourani VH, et al. Transcatheter aortic-valve replacement with a Balloon-Expandable valve in low-risk patients. N Engl J Med 2019;380:1695–705. 10.1056/NEJMoa1814052 30883058

[2] Mylotte D, Andalib A, Thériault-Lauzier P, et al. Transcatheter heart valve failure: a systematic review. Eur Heart J 2015;36:1306–27. 10.1093/eurheartj/ehu388 25265974

[3] Yanagisawa R, Hayashida K, Yamada Y, et al. Incidence, predictors, and mid-term outcomes of possible leaflet thrombosis after TAVR. JACC Cardiovasc Imaging 2017;10:1–11. 10.1016/j.jcmg.2016.11.005 28017712

[4] Capodanno D, Petronio AS, Prendergast B. “Standardized definitions of structural deterioration and valve failure in assessing long-term durability of transcatheter and surgical aortic bioprosthetic valves: a consensus statement from the European Association of Percutaneous Cardiovascular Interven,”. Eur Heart J 2017;38:3382–90. 29020344 10.1093/eurheartj/ehx303

[5] Vollema EM, Kong WKF, Katsanos S, et al. Transcatheter aortic valve thrombosis: the relation between hypo-attenuated leaflet thickening, abnormal valve haemodynamics, and stroke. Eur Heart J 2017;38:1207–17. 10.1093/eurheartj/ehx031 28369242

[6] Dangas GD, Tijssen JGP, Wöhrle J, et al. A controlled trial of rivaroxaban after transcatheter aortic-valve replacement. N Engl J Med 2020;382:120–9. 10.1056/NEJMoa1911425 31733180

